# EANM guideline for harmonisation on molar activity or specific activity of radiopharmaceuticals: impact on safety and imaging quality

**DOI:** 10.1186/s41181-021-00149-6

**Published:** 2021-10-09

**Authors:** Gert Luurtsema, Verena Pichler, Salvatore Bongarzone, Yann Seimbille, Philip Elsinga, Antony Gee, Johnny Vercouillie

**Affiliations:** 1 Department of Nuclear Medicine and Molecular Imaging, University of Groningen, University Medical Centre Groningen, Groningen, The Netherlands; 2 Department of Pharmaceutical Sciences, Medical University of Vienna, Vienna, Austria; 3 Department of Imaging Sciences, King’s College London, London, UK; 4 Department of Radiology and Nuclear Medicine, Erasmus MC, University Medical Centre Rotterdam, Rotterdam, The Netherlands; 5 UMR 1253, iBrain, University of Tours, Tours, France

**Keywords:** Molar activity (A_m_), Specific activity (A_s_), Tracers, Standardisation, Carrier, Mass, Radioactivity, Radiochemistry

## Abstract

This guideline on molar activity (A_m_) and specific activity (A_s_) focusses on small molecules, peptides and macromolecules radiolabelled for diagnostic and therapeutic applications. In this guideline we describe the definition of A_m_ and A_s_, and how these measurements must be standardised and harmonised. Selected examples highlighting the importance of A_m_ and A_s_ in imaging studies of saturable binding sites will be given, and the necessity of using appropriate materials and equipment will be discussed. Furthermore, common A_m_ pitfalls and remedies are described. Finally, some aspects of A_m_ in relation the emergence of a new generation of highly sensitive PET scanners will be discussed.

## Rationale

Harmonisation and standardisation of radiopharmaceutical terms and conventions are necessary to allow radiopharmaceutical research to be unambiguously understood and methodologies tested and transferred between laboratories. The aim of this guideline on molar activity is to provide a review of basic concepts, practical considerations, pitfalls and recommendations for obtaining high molar activity (A_m_) or specific activity (A_s_).

In the field of radiopharmaceutical sciences, small molecules, peptides and macromolecules radiolabelled for diagnostic and therapeutic applications are defined as radiolabelled compounds where a radionuclide is attached by covalent or ionic bonds to a non-radioactive molecular scaffold (precursor). A radiotracer is a radiolabelled compound that is often administered to a complex environment (e.g. a tissue or living organism) without perturbing the system under study.

Most radionuclides used for the production of radiotracers are obtained via ‘no-carrier’ added (n.c.a.) nuclear reactions (definitions for carrier and n.c.a. can be found in Ref. Coenen et al. 2017). However, fluctuations in the amount of carrier contamination from external sources and starting materials during the radiosynthesis can dilute the radiolabelled compound with its non-labelled analogue and thus decrease the A_m_ or A_s_ of the product. Fluctuations in A_m_ or A_s_ for different radiotracer productions may influence the kinetics and tissue uptake of the radiotracers and therefore the quality of the scan data. Increased mass administration can, for example, lead to pharmacological or toxicological effects. Therefore, it is important to describe in detail how A_m_ and A_s_ are defined and to provide appropriate guidelines on how to measure it. Moreover, the overall goal is to introduce a quality standard for A_m_ and A_s_ to standardise and harmonise the measurement and applicability within the radiochemical and pharmaceutical scientific community. Finally, this guideline provides some working examples and recommendations.

## Definition of molar activity (A_m_) and specific activity (A_s_)

A_m_ is defined as the amount of radioactivity per unit mole of the element or compound, is expressed in *Bq/mol* or *GBq/µmol* and is used where the molecular weight of the labelled material is known. A_s_ is expressed in *Bq/g* or *GBq/µg* and is normally used for macromolecules (proteins and antibodies) when the molecular weight of the labelled material is unknown.

Precursor residues or other impurities may be present in a final radiopharmaceutical preparation thus the term apparent molar or specific activity (apparent A_m_ and apparent A_s_) was introduced (Coenen et al. 2017). Apparent A_m_ and apparent A_s_ take into account the amount of non-radiolabelled, radiolabelled impurities and remaining precursor which may interfere with the mass determination of the parent radiotracer.

The additional terms “effective A_m_ and effective A_s_” address other (unknown) materials present in a prepared batch, which have the chemically, biological or pharmacological potential to compete with the radiotracer binding to the intended target. An additional bioanalytical method has to be included to determine the efficacy of a formulated batch, such as receptor or enzyme-binding assays.

## Measurement of molar activity and specific activity.

Molar activity (A_m_) is the ratio between the amount of radioactivity of a solution containing a radiotracer (*Activity*_A*_) and the sum of the quantity of the radioactive compound (n_A*_) plus the quantity of the corresponding isotopically stable compound (n_A_). The unit of A_m_ is typically *Bq/mol* or *GBq/µmol.*

1 $$A_{m} = \frac{{Activity_{{A^{*} }} \left( {GBq} \right)}}{{n_{A} \left( {\mu mol} \right) + n_{{A^{*} }} \left( {\mu mol} \right)}} \simeq \frac{{Activity_{{A^{*} }} \left( {GBq} \right)}}{{n_{A} \left( {\mu mol} \right)}}$$

Here, the $Activity_{A*}$ is the measure of radioactivity present in an exact volume of a formulation measured in becquerel (Bq) using an activity meter (e.g. dose calibrator) (Eq. 1). $n_{A}$ is the quantity of the isotopically stable compound whereas $n_{{A^{*} }}$ is the quantity of the radioactive compound in the same volume. For the determination of the quantity of $n_{A}$, a validated analytical method is required. A reliable and robust HPLC method for the determination of A_m_ or A_s_ includes the validation of the following parameters, as described by the ICH guidelines for analytical methods: accuracy, precision (repeatability), specificity, detection limit, quantification limit, linearity (calibration curve) and range (Balaram et al. 2011). High-performance liquid chromatography (HPLC) or ultra-performance liquid chromatography (UPLC) and a suitable detection system such as UV–Vis spectrometry are generally used. Other analytical tools include, for example, gas chromatography/mass spectrometry (GC/MS) or liquid chromatography/mass spectrometry (LC/MS). See also the EANM guideline on validation of analytical methods for radiopharmaceuticals for more information. https://ejnmmipharmchem.springeropen.com/articles/10.1186/s41181-019-0086-z/.

A calibration curve has to be set up in an appropriate range in order to measure the required amount of compound and should cover concentrations down to the limit of detection (LOD). In principle, the area under the curve (AUC) of, for example, a UV–Vis absorbance (chromatographic peak) describes the sum of the amount of the isotopically stable compound and radiolabelled compound, as the radiolabelled and isotopically stable compound share the same absorption behaviour. However, the quantity of radiolabelled compound ($n_{{A^{*} }}$) changes over time following decay of the concerned radionuclide. Assuming that the quantity of the radioactive compound ($n_{{A^{*} }}$) is per se usually insignificant compared to the associated total moles of analogous stable compound, the sum $n_{A} + n_{{A^{*} }}$ is simplified to be equal to $n_{A}$ (Eq. 1).

**Fig. 1:**
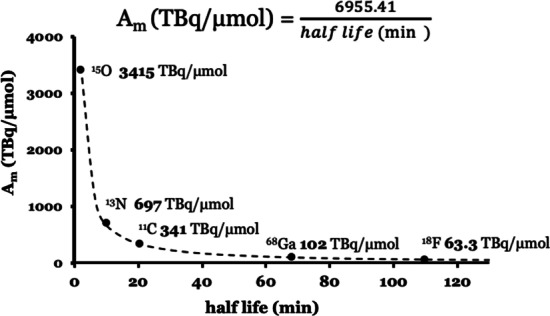
The maximum theoretical molar activity measured in TBq/µmol for PET radionuclides ^15^O, ^13^N, ^11^C, ^68^Ga and ^18^F

The maximum theoretical A_m_ for PET radionuclides ^15^O, ^13^N, ^11^C, ^68^Ga and ^18^F is directly linked (inversely proportional) to their half-life, see Fig. 1. Radionuclides with longer half-lives have lower theoretical A_m_ compared to shorter lived radionuclides_._

Specific activity (A_s_) is usually expressed as *Bq/g* or *GBq/µg* and can be calculated with the same procedure as described for A_m_ having the $Activity_{A*}$ measured in an exact volume of a formulation and the compound measured in *µg* or *g* in the same volume. $m_{A}$ is the quantity of the isotopically stable compound whereas $m_{{A^{*} }}$ is the quantity of the radioactive compound. Assuming that the quantity of the radioactive compound ($m_{{A^{*} }}$) is per se usually insignificant compared to the associated total mass of analogous stable compound, the sum $m_{A} + m_{{A^{*} }}$ is simplified to be equal to $m_{A}$.

A_s_ can also be obtained by dividing A_m_ by the molecular weight (MW—g/mol) of the compound, if this is known (Eq. 2).

2 $$A_{s} = \frac{{Activity_{{A^{*} }} \left( {GBq} \right)}}{{m_{A} \left( {\mu g} \right) + m_{{A^{*} }} \left( {\mu g} \right)}} \simeq \frac{{Activity_{{A^{*} }} \left( {GBq} \right)}}{{m_{A} \left( {\mu g} \right)}} = \frac{{A_{m} \left( {GBq/\mu mol} \right)}}{MW}$$

### Apparent molar activity

The apparent molar activity (apparent A_m_) takes into account the amounts of the radiolabelled (compound A*) and non-radiolabelled radiotracer (compound A), radiolabelled impurities (compound B*) and non-radiolabelled impurities (compound B) and remaining precursor (compound C) present expressed in mol (or *µ*mol). Apparent A_m_ is expressed as *Bq/mol* or *GBq/µmol*.

3 $$\begin{aligned} Apparent A_{m} & = \frac{{Activity_{{A^{*} }} \left( {GBq} \right) + Activity_{{B^{*} }} \left( {GBq} \right)}}{{n_{A} \left( {\mu mol} \right) + n_{{A^{*} }} \left( {\mu mol} \right) + n_{B} \left( {\mu mol} \right) + n_{{B^{*} }} \left( {\mu mol} \right) + n_{C} \left( {\mu mol} \right)}} \\ & \simeq \frac{{Activity_{{A^{*} }} \left( {GBq} \right) + Activity_{{B^{*} }} \left( {GBq} \right)}}{{n_{A} \left( {\mu mol} \right) + n_{B} \left( {\mu mol} \right) + n_{C} \left( {\mu mol} \right)}} \\ \end{aligned}$$

The amount of $n_{A} ,$ $n_{B}$ and $n_{C}$ are determined by a quantitative analysis using calibration curves for A, B and C. The amount of unknown impurities ($n_{B}$) can be difficult to quantify and therefore calculated A_m_ values may be somewhat uncertain. In practice, the unknown impurities are calculated using the absorption coefficient of the intended tracer.

For high quality radiotracer production, it is often mandatory to obtain radiochemical purities ≥ 95% and therefore radiochemical impurities should play a minor role in practice for the calculation of the apparent A_m_. However, non-radioactive scaffolds in routine production represent a separation problem, as for example precursors and peptides with chelators occupied with non-radioactive metal ions share similar retention times.

An different approach of A_m_ calculation specific for radio-metals like gallium-68 labelled compounds is published by the IAEA “Gallium-68 cyclotron productions/TECDOC-1863 (annex 1 pp. 43–45)”. https://www.iaea.org/publications/13484/gallium-68-cyclotron-production

### Description of a procedure to calculate the A_m_ for a carbon-11 radiotracer using an HPLC system equipped with a UV detector

As a first step, the calibration curve is built by developing a validated HPLC method considering the following parameters: UV absorption characteristics of the analyte, mobile phase, column temperature, column pressure and injection volume.

The calibration curve has to consist of at least five different concentrations (in triplicate) ranging from a concentration higher than expected for the radiotracer down to the limit of quantification (LOQ). Plotting the AUCs (*y*-axis) *versus* compound concentration (µmol/mL, *x*-axis) and subsequent linear regression analysis requires a high R^2^-value in order to prove the linearity of the HPLC method. Our recommendation is a R^2^-value > 0.95. The injection of the formulation containing the radiotracer, allows the determination of AUCs from which compound concentrations (µmol/mL) are derived using the calibration curve. When the amount of radioactivity of an exact volume of formulation containing the radiotracer is determined in a dose calibrator (*GBq/mL*), this value has to be corrected for radiochemical purity. That means, only the collected radioactivity corresponding to the product should be included in the calculation.

During method validation, it is crucial to inject the crude mixture from the radiotracer synthesis to assure that (radio)impurities can be identified and baseline separated from the product to enable correct quantification.

Molar activity is not a constant value and changes over time. Therefore, the activity can be decay corrected to a defined reference time point e.g. end of synthesis (EOS) or end of bombardment (EOB). However, for patient or animal application and dose calculation it is a necessity to calculate the molar activity at the time point of administration.

Alternatively, if the mass limit is known, the maximum injectable mass can be specified based on volume i.e. “do not administer more than X mL”. Using this strategy is not necessary to calculate the A_m_ at injection time. However, is it important that the volume of the end product is recorded accurately. Of course, consultation with the end users is crucial before embarking on a scanning campaign, so that all these issues can be agreed in advance of the scanning sessions.

**Fig. 2:**
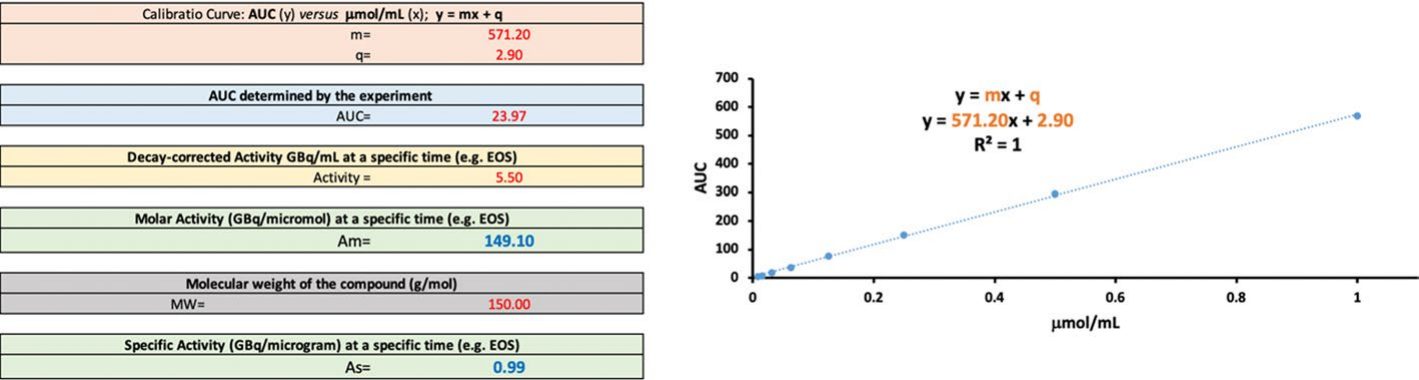
A_m_ and A_s_ calculation using the molar activity calculator

*Example of how to calculate A*_*m*_* and A*_*s*_* using Excel*: Having a calibration curve of y = 571.20 x + 2.90 and a solution with an AUC of 23.97, the non-radioactive compound concentration present in the formulation is 0.04 µmol/mL. With an *Activity*_*A*_ of 5.50 GBq/mL at EOS, the A_m_ at EOS is 149.1 GBq/µmol. The A_s_ (GBq/µg) is derived from A_m_ by dividing by the molecular weight of the compound (Fig. 2, MW = 150, A_s_ = 0.99 GBq/µg at EOS).

Important in the context of A_m_ calculation is the limit of quantification (LOQ) as evaluated during the method validation process. In radiochemistry it is common, that product concentrations below the LOQ are measured at the end of radiosynthesis. Here, the most correct value for product amount is to use the LOQ for the calculation instead of extrapolation of the data, followed by the statement that ‘A_m_ is greater or equal to the calculated value’. An extrapolation beyond the linearity and limitation of an analytical method used for the calculation of the mass or molar concentration of the final product may overestimate the respective A_s_ and A_m_ and must be avoided.

## Examples of variability of molar activities for clinically applied PET radiotracers in different production laboratories

In general, molar activities play a crucial role for all PET radiotracers targeting saturable binding sites (e.g. receptors) but are secondary or negligible for many metabolic PET radiotracers where the endogenous levels of the compound are present in great excess of the radiotracer itself. The fluctuation in A_m_ for carbon-11 labelled compounds is mainly caused by contamination with varying amounts of carrier carbon. A consistent small amount will come from the target gas but ingress of atmospheric carbon dioxide into the cyclotron target cannot be totally precluded and this will reduce the A_m_ of the radiosynthon [^11^C]CO_2_ or [^11^C]CH_4._ Small amounts of carrier may also come from target body materials, valves, tubing etc. Similar challenges exist when processing radiometals and their contamination by non-radioactive metal contaminants. The examples below focus on carbon-11 PET radiotracers for comparison of A_m_ derived from different laboratories.

**Fig. 3:**
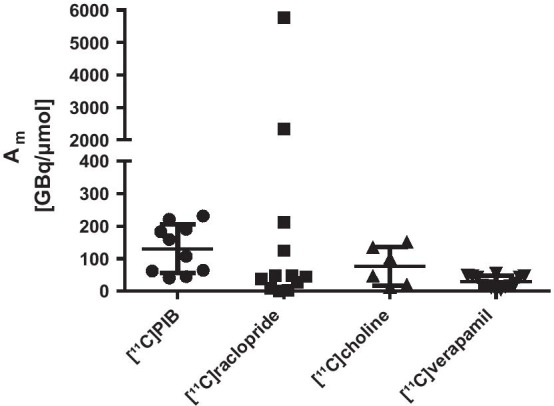
Distribution of A_m_ as described in literature for the clinically applied PET-tracers [^11^C]PIB, [^11^C]raclopride, [^11^C]choline and [^11^C]verapamil produced at different PET-centres

A_m_ varies between laboratories and radiotracers as exemplified in Fig. 3 for the PET radiotracers [^11^C]PIB (Philippe et al. 2011; Coliva et al. 2015; Buccino et al. 2019; Zhou et al. 2007; Verdurand et al. 2008; Boudjemeline et al. 2017), [^11^C]raclopride (Kawashima et al. 2015; Ishiwata et al. 1999, 2001; Oswald et al. 2005; Wilson et al. 2000; Andersson et al. 2009; Lee et al. 2016; Fujimura et al. 2010; Volkow et al. 2008; Alexoff et al. 2003; Schiffer et al. 2005; Farde et al. 1986; Mauger et al. 2005), [^11^C]choline (Biasiotto et al. 2012; Rosen et al. 1985; Cheung and Ho 2009; Reischl et al. 2004; Hara et al. 1997; Hara and Yuasa 1999) and [^11^C]verapamil (Elsinga et al. 1996; Hendrikse et al. 1998, 2001; Ikoma et al. 2006; Klerk et al. 2009; Assema et al. 2012; Wagner et al. 2011; Wegman et al. 2002; Luurtsema et al. 2003, 2005; Sasongko et al. 2005; Lubberink et al. 2007; Nagatani et al. 2001; Takano et al. 2006). Based on this literature, the common range for molar activities is 1 to 230 GBq/µmol (calculated at EOS) applying the ‘wet’ or gas phase method of [^11^C]methyl iodide production. The variation of A_m_ are higher for [^11^C]PIB and [^11^C]choline than for [^11^C]verapamil. [^11^C]Raclopride, in this example, is an exception as two particularly high molar activities are found in literature. It is stated, that these higher molar activities can be achieved by applying a [^11^C]methane cyclotron target (Andersson et al. 2009; Fujimura et al. 2010; Noguchi and Suzuki 2003).

## Examples highlighting the importance of high A_m_

### Examples of highly toxic molecules: [^11^C]carfentanil, (+)-[^18^F]flubatine and (+)-[^11^C]PHNO

[^11^C]Carfentanil is a PET radiotracer used for imaging studies of µ-opioid receptors. The radiotracer is widely used to investigate the role of the of µ-opioid system in relation to obesity, pain regulation, drug addiction and many other disorders (Blecha et al. 2017). Carfentanil is approximately a thousand times more potent than morphine (Daele et al. 1976) Even at very low doses, unlabelled carfentanil can elicit a pharmacological response. Therefore, the mass limit is set to ≤ 0.03 µg/kg as an accepted standard (Blecha et al. 2017). This means that, for a clinical PET scan of an average subject of 70 kg, the calculated maximum administered mass is 2.1 µg (5.3 nmol). A high A_m_ of [^11^C]carfentanil is therefore required to use this radiotracer safely. An injection dose of 400 MBq with 2.1 µg of carrier, can only be achieved if the A_m_ of > 75 GBq/µmol at injection time (Perkins et al. 2014).

Many groups have developed radioligands targeting α4β2 as they are involved in various CNS disorders, Alzheimer’s and Parkinson’s neurodegenerative diseases, Tourette syndrome, anxiety, pain and depression. Development of PET radiotracers were mostly based on the epibatidine scaffold, which displays high toxicity (Mu et al. 2006). Structural modulations have been performed to improve affinity and selectivity in order to reduce toxicity. Nevertheless, these compounds still display toxic effects. For example, (+)-flubatine has a NOEL (no observed effect level) of 1.55 µg/kg in humans (Mu et al. 2006). The NOEL value requires the administration of (+)-[^18^F]flubatine with a A_m_ exceeding 700 GBq/µmol in order to maintain a 1000-fold safety margin for the use of the radiotracer in clinical studies (Smits et al. 2014).

The potent dopamine D_2_/D_3_ receptor agonist (+)-PHNO ((+)-4-propyl-3,4,4a,5,6,10*b*-hexahydro-2*H*-naphtho[1,2-*b*][1,4]oxazin-9-ol, molecular weight 247.33 g/mol) can cause nausea, vomiting and orthostatic hypotension, at doses of 0.25 mg three times a day (Weiner et al. 1989). In initial clinical studies using the radiotracer (+)-[^11^C]PHNO, participants commonly experienced nausea (Willeit et al. 2006, 2008; Chiuccariello et al. 2013). Houle and co-workers subsequently recommended a dose below 0.029 µg/kg for (+)-[^11^C]PHNO, and therefore a maximum administered dose of 2.03 µg for a 70 kg participant, which is even lower than for [^11^C]carfentanil (Blecha et al. 2017). In this case, a molar activity of > 48.5 GBq/µmol at time of injection is required to avoid any side effects.

### Example for reduced image quality based on apparent molar activity: [^11^C]***m***HED

Metaraminol, the precursor of the radiotracer [^11^C]*meta*-hydroxyephedrine ([^11^C]*m*HED (Mizrahi et al. 2009)) is authorised for the treatment of acute hypotension (Rosenspire et al. 1990). Both metaraminol as well as *m*HED itself can lead to increase of both systolic and diastolic blood pressure, therefore the sum of both compounds has to be evaluated after purification of the radiotracer and considered for safe administration of [^11^C]*m*HED (apparent A_m_) (Khavandi et al. 2009). Doses of 0.25–1 mg of metaraminol (Vraka et al. 2019) were safe to inject to patients. In most PET measurements, a pharmacological effect must be avoided and therefore a dose below a pharmacological or even toxic dose is a necessity (microdosing concept) (Critchley et al. 1999). In literature, the NOEL for *m*HED is not described, therefore it is recommended to include a safety margin for the calculation of the maximal applicable dose for a PET application. If calculated with e.g. a safety margin of 100 for the administration of [^11^C]*m*HED a maximal absolute dose of 2.5–10 µg (sum of *m*HED and metaraminol) per administration can be used as a limit to avoid any pharmacological effects during a PET-scan. Even at non-toxicological doses of *m*HED and metaraminol, it has been reported that doses of approximately 50 nmol/kg result in a decrease in myocardial uptake in mice (Bergström et al. 2003). This can be attributed to saturation of the norepinephrine receptors in the myocardium (competition between [^11^C]mHED and non-labelled mHED and metaraminol), and thus is a good example of the importance of maintaining high molar activities and apparent molar activities.

## Examples highlighting where high A_m_ is less important or detrimental

### [^18^F]FDG and labelled endogenous molecules

Endogenous molecules, like amino acids, fatty acids, choline and glucose, labelled with radionuclides, undergo competition with the corresponding endogenous compounds in blood/tissue. Therefore, high A_m_ is not usually required for these radiotracers. Although [^18^F]FDG is not an endogenous molecule, its uptake is related to the glucose levels in plasma. [^18^F]FDG with low A_m_ does not result in deterioration of the PET image quality. However, high blood glucose levels do influence the radiotracer behaviour significantly, requiring strict adherence to fasting regimen for patients undergoing [^18^F]FDG scans (Law et al. 2010).

### [^111^In]J591 → low A_m_ is required

If A_m_ plays a role in terms of pharmacological or even toxic effects, image quality has to be verified for each and every radiotracer. Especially for antibodies, it has been reported that the administration of [^111^In]J591 with low A_m_, achieved by adding unlabelled antibody, benefits the residence times and lesion to non-lesion residence time ratios (Trifiro and Paganelli 2007). The study suggests that higher administered antibody masses saturate the liver uptake and thus increase the biological half-life of the radiotracer. However, no standardised studies are yet available investigating the correlation of A_m_ and image quality (signal-to-noise, standardized uptake value—SUV) for common radiotracers, requiring further research.

### [^18^F]FDOPA → conflicting results- discussion about the role of A_m_

**Fig. 4:**
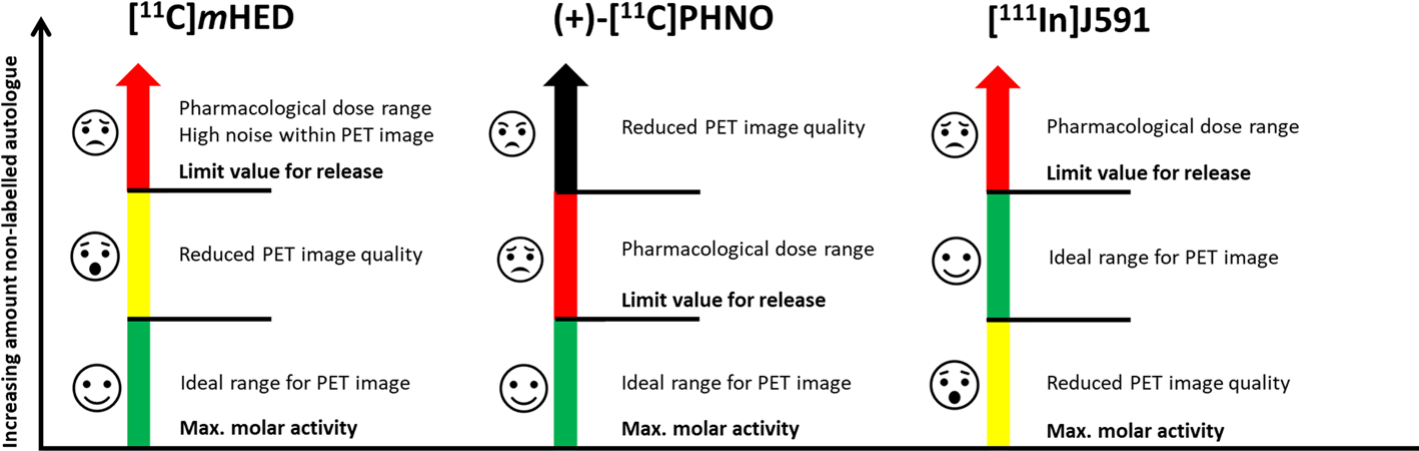
Examples displaying the connection of molar activity, PET image quality and pharmaceutical dose. (left) [^11^C]mHED, requires high A_m_ for sufficient quality of the PET scan. Before a pharmacological dose is reached the image quality is affected. (middle) [^11^C]PHNO requires high A_m_ for patient application and safety. At first a pharmacological effect, like nausea, is observed before the quality of the PET image is influenced. The release criteria has to be strictly followed for this kind of compounds to avoid any side or toxic effects. (right) [^111^In]J591 is reported to have a low lesion to non-lesion residence time if the molar activity is high. Higher amounts of non-labelled autologue increases the residence time and subsequently the quality of the image

6-[^18^F]fluoro-3,4-dihydroxy-L-phenylalanine ([^18^F]FDOPA) has clinical indications in neurology and oncology. Kuik et al. (Pandit-Taskar et al. 2009) reported in a neuroendocrine tumour model study the use of [^18^F]FDOPA administered at doses spanning 3 orders of magnitude (11–33,000 GBq/µmol). In this study, the authors reported no significant difference in mean SUV and no statistically significant differences between the uptake values in biodistribution experiments between high and low A_m_ groups. In conclusion, the authors claimed that even a 3000-fold difference in A_m_ produced no significant differences in signal. Nevertheless, a similar clinical study using very high A_m_ [^18^F]FDOPA (4000 GBq/µmol), showed higher uptake values compared to previous studies with significantly lower A_m_ (Kuik et al. 2015). The authors indicated that the increased striatal-to-occipital-cortex ratio (SOR) observed could have been due to either the higher spatial resolution of the PET/CT scanner used, or the higher A_m_ of the radiotracer in comparison to the previous studies. In addition to the potential effect on the image quality, low A_m_ [^18^F]FDOPA can cause carcinoid crisis and flushes (Akamatsu et al. 2017 Feb) (Fig. 4).

## Influence of materials and beam parameters on A_m_ and A_s_

The selection and the use of appropriate materials and optimal beam performance are important to obtain high A_m_ and A_s_. Below is a list of critical items which may have an influence on A_m_ and A_s._

- Quality of irradiated starting material. The production of most common radionuclides is performed using a cyclotron equipped with specially designed targets filled with starting materials. It is evident that staring materials must be highly pure to obtain high A_m_ and A_s_. Impurities and carriers can influence A_m_ and A_s_.
- Quality of gasses: Gas targets are used for the production of [^11^C]CO_2_ or [^18^F]F_2_ and filled with highly pure gas (99.999%) mixture of N_2_ + O_2_ (0.05–2%) or Ne or [^18^O]O_2_, respectively.
- Quality of liquids: For the production of [^18^F]NaF liquid targets are used, filled with [^18^O]H_2_O. Reusing of the target water can reduce the A_m_.
- Quality of generators: For generator-based radiometals the frequency of elution affects the apparent A_m_, as over the time the amount of the granddaughter decay product increases, e.g. ^68^Zn for ^68^ Ga/^68^Ge generators.
- Other parameters such as, irradiation time and beam current can also have influence on A_m_ and A_s_. For radiometals, these parameters are dependent on the producer of the radiometal or generator and cannot by influenced by the radiochemistry method per se.
- Target maintenance and cleaning. See for procedures in IAEA document https://www-pub.iaea.org/MTCD/Publications/PDF/Pub1563_web.pdf

## Recommendations to obtain high A_m_ and A_s_

Several recommendations about target gas, columns to trap contaminations, target handling and general comments to support high A_m_ and A_s._ are listed in Table 1.

**Table 1:** Recommendations to obtain high A_m_ in C-11 and F-18 productions

Production of carbon-11	General comments
Use highly pure target gas 6.0	Use only highly pure target gas and flush gas (purity: 6.0, 99.9999%) to avoid carrier CO_2_ in target. Via isotope exchange in target the A_m_ of the final product will decrease. The target gas mixture should be analysed for carbon < 0.1 volume per million (VPM) of CO_2_ and CO (detection limit). The use of gas purification cartridges may also be advantageous. Low volume targets are to be preferred to obtain high A_m_/A_s_
Use highly pure target flush gas 6.0 (Helium, Nitrogen or Argon)	Target flush or push gas transports [^11^C]CO_2_ from target to synthesis module. Use only highly pure target flush gas 6.0 (99.9999%) to avoid non-radioactive CO_2_ accumulation in the CO_2_ trap. Note: if Argon is used as push gas beware it condenses at − 150 °C
Insert a molecular sieve (CO_2_ trap) in line before target	Most suitable inline CO_2_ trap is an Ascarite® column. CO_2_ will be converted to carbonate under basic conditions and sticks in the column. This process can influence the performance of the column and therefore important to replace the column frequently
Prefer inert tubing to the target and from target to hotcell (stainless steel, PEEK)	The choice for the appropriate inert material, depends on the situation of the lab. For instance, PEEK tubing must be replaced frequently and if stainless steel tubing’s are used, there must be a possibility for frequent rinsing of these lines to remove carrier carbon
Use inert target and target seals	Carrier carbon could be released and migrate from target metal and seals into target body
Fill the target directly after irradiating with target gas	The target must be filled to avoid isotope exchange of carbon with highly pure target gas from the surrounding area
Dump irradiation each morning	Because of possible minor leaks into the gas handling system, like valves and tubing, the first irradiation provides lower A_m_ compared to the following irradiations. Keep in mind that air contains 380 ppm carbon dioxide. It can be advantageous to flush the target and tubing to the radiosynthesis system several times with high purity gas (e.g. helium) to remove carrier carbon
Use of glovebox for solvent storage and dissolving precursor	Glove box filled with highly pure gas (preferred to use argon) avoids accumulation of CO_2_ in the solvent used to dissolve precursors

Production of fluorine-18	General comments
Use highly pure enriched water ([^18^O]H_2_O) and check certificate of analysis (COA) of enriched water for fluoride content	To avoid trace of carrier cold fluoride (^18^F is diluted with ^19^F), and will decrease the A_m_
Use highly pure target push gas 6.0 (Helium, Nitrogen or Argon)	To avoid trace of carrier fluoride. See comments in previous table
Use inert tubing from target to hot cell (like PEEK, PTFE)	To avoid trace of carrier fluoride. See comments in previous table above. Selection of inert tubings must be done carefully and the selection of materials are site specific
Clean target	Clean according to an approved cleanings procedure to avoid possible accumulation of carrier fluoride in target. See for procedures in IAEA document https://www-pub.iaea.org/MTCD/Publications/PDF/Pub1563_web.pdf
Radiotracer production	Notice that a radio-synthesis starting with [^11^C]CO_2_ and using reagents that are reactive at ambient temperatures, often yield products with low A_m_. E.g. radiosynthesis with Grignard reagents and organolithium compounds
Use inert materials for cassettes, tubing, valves etc.	Selection of inert materials must be done carefully and the selection of materials are site specific
Use pure precursors (99%)	To obtain radiotracers with high A_m_ it is important to avoid traces of carrier during the synthesis
Use high purity chemicals and gasses (e.g. acetonitrile, potassium carbonate, Helium, Nitrogen etc.)	See above
Use minimising amounts of starting materials/reagents	Minimising added carrier as contaminants could be beneficial to obtain high A_m_

## Pitfalls related to carbon-11 radiotracer production (what to do when introduction of carrier occurs?)

- Check and analyse target gasses for carrier
- Check leakage in the [^11^C]CH_3_I system. Air contains CO_2_ (380 ppm)
- Check precursor and chemicals for carrier
- Optimise synthesis times to increase radiochemical yield
- Optimise preparative HPLC method to avoid traces of labelling precursors and other impurities in the final product.

## Radiometals and A_m_

Over the last two decades, an increasing number of radiotracers have been labelled with radiometals for the personalized management of cancer patients. The radiotracers are usually labelled with an imaging radionuclide for the diagnosis, dosimetry and treatment planning, followed by labelling with a beta- or alpha-emitting radionuclide (e.g. ^177^Lu or ^225^Ac) for therapeutic applications. Such an approach corresponds to the so-called ‘theranostic’ paradigm.

In this section, we emphasized on gallium-68, however the following considerations can also be applied to other radiometals. Gallium-68 is increasingly used for labelling small biomolecules, such as peptides and small antibody fragments, and it is one of the main generator-based positron-emitting radionuclides currently used for PET imaging. ^68^ Ga is therefore particularly convenient because its production does not require an on-site cyclotron. Moreover, its 68 min half-life matches the rapid uptake kinetics of most radiolabelled peptides. Additionally, the ‘short’ half-life of gallium-68 and the rapid clearance of the radiolabelled peptides provide a suitably ‘low’ radiation dose to the patient. Consequently, several ^68^ Ga-labelled peptides have been developed to image various targets in tumour lesions, such as the somatostatin receptor subtype 2 ([^68^ Ga]Ga-DOTA-TATE), the prostate specific membrane antigen ([^68^ Ga]Ga-PSMA-617), the chemokine receptor type 4 ([^68^ Ga]Ga-Pentixafor), the gastrin releasing peptide receptor ([^68^ Ga]Ga-NeoBOMB1) and integrin α_v_β_3_ ([^68^ Ga]Ga-NOTA-PRGD2) (Koopmans et al. 2005).

Gallium-68 complexation chemistry has been studied extensively and several bifunctional chelators are commercially available allowing stable ^68^ Ga-chelator complexes and conjugation to the peptide at the terminus position or on a specific functionality of amino acid side chain (i.e. lysine, cysteine) (Tanzey et al. 2018). Radiolabelling conditions have been optimized to warrant the robustness of the ^68^ Ga-labelling (Price and Orvig 2014). Indeed, the radiotracer preparation is generally fast and the incorporation of gallium-68 is nearly quantitative. Separation of the final radiotracer from the peptide precursor or the radionuclide is typically avoided during the radiochemical process. However, in the scenario of an incomplete radiometal incorporation, a simple purification of the radiotracer via solid phase extraction can be carried out to remove unreacted radionuclide. The main factor influencing the A_m_ of a radiometallated peptide is the presence of peptide precursor and metal ion contaminants. The latter can compete with gallium-68 during the complexation reaction with the chelator. Thus, metal ion contaminants indirectly affect A_m_ by necessitating higher amounts of peptide precursor to achieve high radionuclide incorporation. Rigorous elimination of metal impurities during the synthesis of the chelator-peptide conjugate, production and isolation of the radionuclide, and the radiolabelling is compulsory to obtain the highest achievable A_m_ (Velikyan 2014). In fact, we are normally referring to the apparent A_m_ for such radiotracers, empirically determined by dividing the amount of radioactivity obtained by the amount of peptide precursor engaged in the labelling process, since the final formulation of the labelled radiotracer contains remaining precursor and peptide complexed with metal impurities.

The requirement for high A_m_ is however dictated by several biological factors, such as the affinity of the peptide for its receptor and the number of available receptors, limiting the mass dose of peptide that can be administered. Indeed, a suboptimal A_m_ could result in excessive amount of peptide leading to the saturation of the receptor or eventually to the induction of pharmacological side effects, as it has been previously observed with GRPR agonists (Velikyan et al. 2008). Besides, the concomitant presence of unlabelled and labelled peptide binding to the same receptor can hamper a sufficient accumulation of radioactivity in the target tissue, and therefore deteriorate the quality of the PET images. Hence, a high A_m_ is of paramount importance when developing PET radiotracers for saturable regulatory peptide binding processes or highly toxic molecules. Nevertheless, there are also situations, such as the somatostatin receptor-positive tumours, where the administration of an optimized dose of radiolabelled peptide is required to improve the sensitivity of detection. Indeed, it has been demonstrated that the uptake in somatostatin receptor-positive tumours is a bell-shaped function of the injected mass of peptide. With a higher administered mass dose of peptide, an increase of the tumour uptake is initially observed, most likely due to a faster rate of internalization by ligand-induced receptor clustering, followed by a drop in the tumour accumulation caused by the saturation of the receptor (Breeman et al. 2011).

In summary, there is no consensus on the A_m_ for radiolabelled peptides, and an optimal value, that could be for instance a compromise between saturable elimination and receptor saturation, has to be experimentally defined.

## A_m_ considerations relating to a new generation of highly sensitive PET scanners: total body

Recent developments in PET scanner design have seen the emergence of a new generation of camera, the ‘total body’ PET scanners. By virtue of their extended detector coverage [up to two metres field of view cf. fifteen centimetres for a ‘typical’ PET-CT scanner these scanners currently report ca. five times greater sensitivity for dynamic imaging applications compared with ‘standard’ scanners (Breeman et al. 2001). The increase in sensitivity achievable with these tomographs may present new opportunities and challenges for the radiochemistry field.

The prospect of being able to administer lower doses of activity in total body PET scanning also implies that the administered radiotracer mass will be much lower. Currently, typical administered masses are often in the 2.5–25 nmol range. The injected mass of small molecule radiopharmaceuticals on classical PET–CT tomograph are normally in the range of 0.1–10 µg.

Assuming a fivefold increase in scanner sensitivity, administered radiotracer masses in the picogram range would be thus routinely achievable. This has several implications: (1) a significant decrease in administered radiotracer mass may enable the detection of poorly expressed molecular recognition sites [e.g. receptors, enzymes] not ‘imageable' using current practice as these molecular targets are significantly occupied by carrier tracer mass; (2) that radiotracers may be administered at a dose below the regulatory 1.5 µg ‘cut-off’ for unknown excipients in tablets and drug formulations (Cherry et al. 2018) requiring no traditional toxicology testing. This may be a way to enable translational studies at ‘sub-toxicological’ doses without the frequently encountered ‘toxicology testing cost barrier’ hampering first-in-human studies of otherwise promising novel radiotracers. If achievable, this could greatly enhance the productivity of new radiotracer evaluation and introduction for human use.

Footnote: Typically—for a small molecule radiotracer with a molecular weight 400, injected activity of 200 MBq and a molar activity of 10 GBq/µmol this corresponds to an injected mass of 8 µg (20 nmol).

For more information about toxicity of tracers, see position paper https://ejnmmipharmchem.springeropen.com/articles/10.1186/s41181-016-0004-6.

## Conclusions

The A_m_ or A_s_ of radiopharmaceuticals are important parameters and has an impact on safety and imaging quality This guideline will assist professionals to correctly define and measure molar activity (A_m_) and specific activity (A_s_).

## Data Availability

The datasets used and/or analyzed during the current study are available from the corresponding author on reasonable request.

## Abbreviations

A_m_: Molar activity
A_s_: Specific activity
Bq: Becquerel
[^18^F]FDOPA: 6-[^18^F]fluoro-3,4-dihydroxy-L-phenylalanine
GC/MS: Gas chromatography/mass spectrometry
HPLC: High-performance liquid
LC/MS: Liquid chromatography/mass spectrometry
NCA: No-carrier added
MW: Molecular weight
NOEL: No observed effect level
PET: Positron emission tomography
SOR: Striatal-to-occipital-cortex ratio
SUV: Standard uptake value
UPLC: Ultra-performance liquid chromatography
